# Helios: A Scalable 3D Plant and Environmental Biophysical Modeling Framework

**DOI:** 10.3389/fpls.2019.01185

**Published:** 2019-10-18

**Authors:** Brian N. Bailey

**Affiliations:** Department of Plant Sciences, University of California, Davis, Davis, CA, United States

**Keywords:** biophysical model, functional-structural plant model, software architecture, terrestrial LiDAR, three-dimensional model

## Abstract

This article presents an overview of Helios, a new three-dimensional (3D) plant and environmental modeling framework. Helios is a model coupling framework designed to provide maximum flexibility in integrating and running arbitrary 3D environmental system models. Users interact with Helios through a well-documented open-source C++ API. Version 1.0 comes with model plug-ins for radiation transport, the surface energy balance, stomatal conductance, photosynthesis, solar position, and procedural tree generation. Additional plug-ins are also available for visualizing model geometry and data and for processing and integrating LiDAR scanning data. Many of the plug-ins perform calculations on the graphics processing unit, which allows for efficient simulation of very large domains with high detail. An example modeling study is presented in which leaf-level heterogeneity in water usage and photosynthesis of an orchard is examined to understand how this leaf-scale variability contributes to whole-tree and -canopy fluxes.

## Introduction

Biophysical processes in plant and environmental systems traverse an extraordinary range of spatial and temporal scales, with high heterogeneity commonly present across these scales. In plant ecosystems, this is particularly true, as important effects of heterogeneity have been frequently reported across the full range of scales from cells up through the globe (e.g., Mott and Buckley, 2000; Valladares, 2003). Often, it is convenient to study plant systems at scales most relevant to humans—leaves to canopies in space and seconds to months in time. Obtaining observations beyond these scales often requires high effort that may yield little additional useful information. However, it is clear that heterogeneity across scales can have significant impacts on exchanges of mass, momentum, and energy, and understanding how heterogeneity augments transport processes is key in understanding links between plant structure and function.

To circumvent limitations in our ability to observe plant systems across the entire range of relevant scales, it is common to use mathematical models to translate information obtained at one scale to another scale of interest where data are lacking. In order to do so, assumptions of homogeneity are typically made over a certain range of scales. The earliest, and still most frequently used, class of models of plant systems assumes homogeneity in horizontal directions, thus effectively treating a plant canopy as a “big leaf” (Sinclair et al., 1976; Raupach and Finnigan, 1988; Amthor, 1994; Friend, 2001). In some cases, homogeneity is assumed in all directions including the vertical, which is convenient because it means that a measurement or model prediction at any point in space can be considered representative of the entire plant system. A class of big-leaf models called “multilayer models” accounts for vertical heterogeneity by limiting assumptions of homogeneity to a discrete vertical level of vegetation (Meyers and Paw U, 1987; Baldocchi and Harley, 1995). As a compromise between the single big-leaf and multilayer approaches, two-leaf models have also been developed that assume that leaves are either sunlit or shaded, thus effectively limiting model calculations to two-leaf layers (DePury and Farquhar, 1997; Wang and Leuning, 1998).

Although assumptions of large-scale homogeneity are convenient in translating observations and understanding across scales, in nature these assumptions are frequently violated. The current generation of plant system models has tended toward a high-resolution, three-dimensional (3D) approach that explicitly resolves heterogeneity in plant structure at scales of individual plants or smaller (Wang and Jarvis, 1990; Pearcy and Yang, 1996; Sinoquet et al., 2001; Allen et al., 2005; Dauzat et al., 2007; Hemmerling et al., 2008; Pradal et al., 2008; Evers et al., 2018). Early 3D models began by discretizing canopies into individual plants, which allows for the representation of heterogeneity in plant shape, size, and arrangement (e.g., Wang and Jarvis, 1990; Cescatti, 1997). Advances in computational power have enabled more detailed models that discretize plants into homogeneous volumes at submeter scales (e.g., Sinoquet et al., 2001; Bailey et al., 2014; Bailey et al., 2016) or models that resolve individual leaves (e.g., Pearcy and Yang, 1996; Allen et al., 2005; Dauzat et al., 2007; Hemmerling et al., 2008; Pradal et al., 2008).

There is no model that is ideally suited for all applications, and each of the models introduced above makes many trade-offs that are suitable for the particular system and phenomena of interest. A few important trade-offs in plant systems models are discussed below:

*Model complexity vs. computational expense*. Increases in model complexity generally incur corresponding increases in computational expense. Simple models like the “big-leaf” approach described above are very computationally efficient, and thus they can be used to simulate extremely large problems such as global ecosystem fluxes (Churkina et al., 2005; Reichstein et al., 2005; Lawrence et al., 2019). However, errors and biases can be sizable if subcanopy heterogeneity plays a significant role in the biophysical processes of interest (Friend, 2001; Ponce de León and Bailey, 2019). Models that resolve plant-level heterogeneity often incur a significant computational cost, but simulations are usually limited to domain sizes with a few dozen large plants (Duursma and Medlyn, 2012; Vezy et al., 2018). Leaf-resolving models incur yet another step increase in cost and usually limit the maximum domain size to a dozen or fewer plants depending on plant size and overall model complexity (Hemmerling et al., 2008; Pradal et al., 2008; Kahlen and Stützel, 2011; Woods et al., 2018).

*Ease of use vs. flexibility*. Providing users with more control over software configuration and execution typically comes with the trade-off of decreasing ease of use (Holzinger, 2005). By automating many tedious or technical tasks, developers can design software that can be readily utilized by inexperienced users. However, for more advanced users who may wish to use the software in ways not originally envisioned by the developers, this can create severe limitations. In the context of plant models, model coupling and execution are often not sequential. For example, if one wishes to simulate photosynthesis of a leaf, this process is cyclically dependent on a number of other processes; photosynthetic rates are dependent on leaf temperature, which is dependent on latent cooling as mediated by stomatal conductance as well as longwave emission, which is also dependent on the leaf temperature. Coupling of the above processes in a model often requires iteration, which can require flexibility if incorporated within a generalized modeling framework. This issue was discussed by Pradal et al. (2008) in the context of the development of the OpenAlea plant modeling framework, which increases ease of use by compromising some flexibility in terms of its execution model. Most 3D plant growth modeling frameworks use a linear work flow in which the execution of various submodules is predefined in order to produce a standard set of outputs (Hemmerling et al., 2008; Pradal et al., 2008; Henke et al., 2016).

Choice of programming language also has important implications in terms of this trade-off. In order to improve ease of use, many modeling frameworks choose to utilize simpler yet less efficient languages such as Python or Java that may not require an explicit compilation step or memory management (Hemmerling et al., 2008; Pradal et al., 2008; Boudon et al., 2012; Henke et al., 2016). Other frameworks have transitioned toward more efficient and flexible languages such as C++ at a sacrifice in usability (Karwowski and Prusinkiewicz, 2003).

*Model complexity vs. availability of input data*. Increasingly complex models require increasingly complex inputs, and often progress in model development outpaces the development of methods for specifying detailed model inputs. In some cases, models originally built on a solid mechanistic foundation can essentially become overfitted empirical models when inputs turn into free parameters that cannot be measured (Ginzburg and Jensen, 2004). Thus, the development of detailed predictive models is frequently limited by the ability to provide them with realistic inputs, and the argument could therefore be made that in some cases simpler models may be more practical (Raupach and Finnigan, 1988).

This work introduces the new 3D plant and environmental modeling framework “Helios,” which is differentiated from other available frameworks in terms of the way in which the above trade-offs are prioritized. First, Helios is a flexible modeling framework that allows for efficient and extensible coupling between arbitrary submodels called plug-ins. Unlike most previous models, it is formulated to allow for maximum control by the user over submodel coupling, execution, and data flow, enabling models with complex feedbacks. However, this comes with a sacrifice in ease of use, as the user often must decide the order and timing of submodel execution. Helios is intended to utilize state-of-the-art biophysical models with high complexity in order to maximize physical realism. In order to afford this high model complexity, many Helios plug-ins perform calculations using graphics processing unit (GPU) hardware, which enables a unique combination of model complexity and range of scales that can be feasibly represented. Finally, Helios includes a plug-in that allows for automatic generation of architectural inputs based on terrestrial LiDAR data.

The goal of this work is to provide a high-level overview of Helios. For specific details regarding implementation and usage, readers are referred to the extensive documentation included with the software.

## Core Engine

### Model Geometry and Data

**Figure 1:**
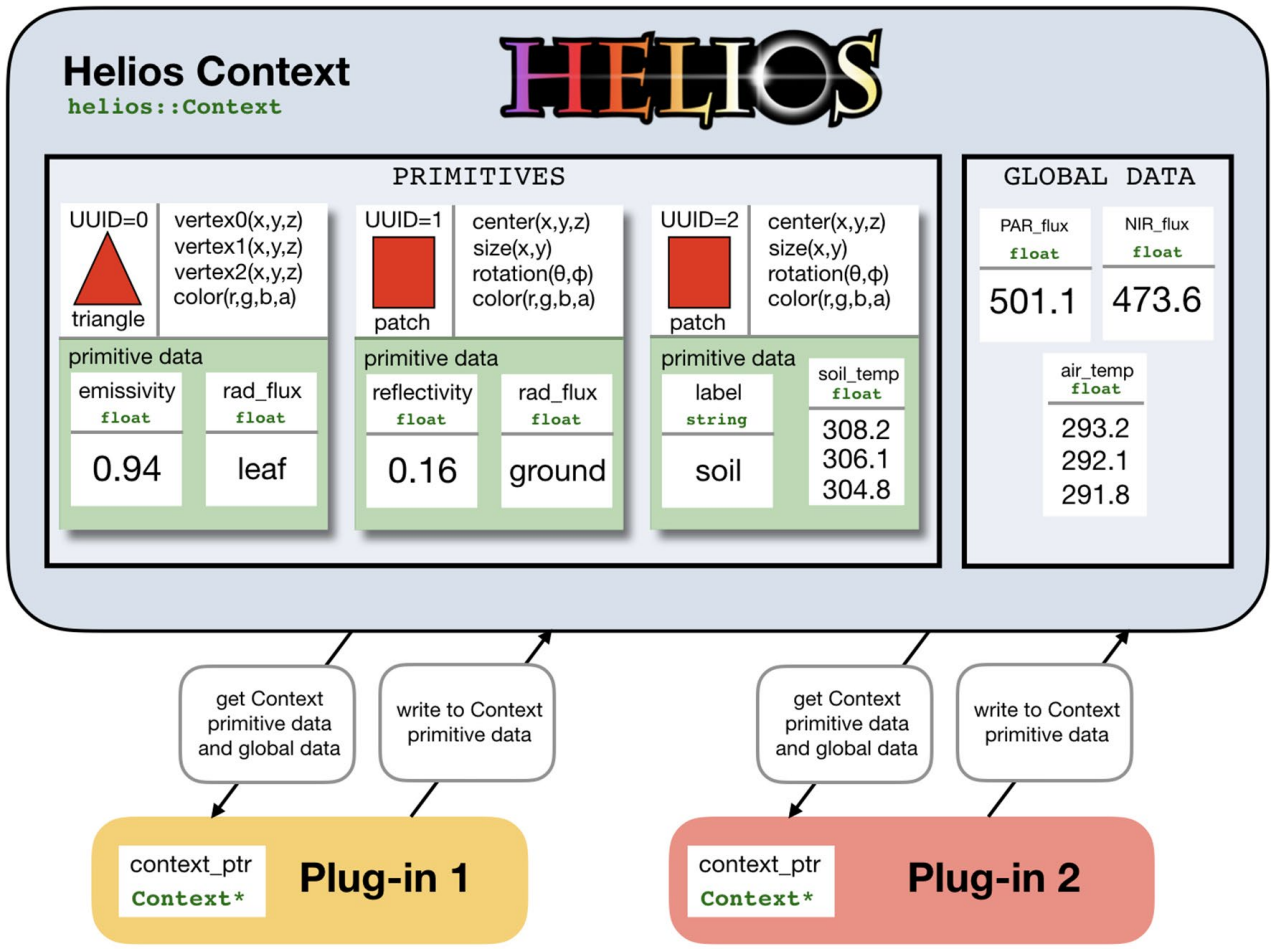
Schematic of data representation and model coupling in Helios. The Context manages model geometry and associated data structures. Model geometry consists of elemental polygons: either triangles, patches (rectangles), or voxels (parallelepiped). Each element may have varying types of associated data called “primitive data,” and there may be additional data structures not mapped to individual elements called “global data.” Model plug-ins are coupled by operating on common data structures within the context.

**Figure 2:**
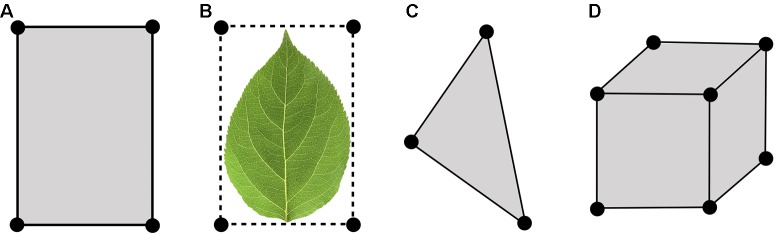
Types of primitive geometric elements available in Helios: **(A)** patch, **(B)** patch masked by an image transparency channel **(C)** triangle, **(D)** voxel.

At the core of Helios is the Context class, which manages model geometry and data (**Figure 1**). Model geometry is formed using three types of primitive elements: triangles, patches (rectangles), and voxels (parallelepiped) (**Figure 2**). Triangles and patches can be masked using the transparency channel of a PNG image file to create planar elements with arbitrary shapes, which is a common approach in both computer graphics applications (Suffern, 2007) and other plant modeling software (Hemmerling et al., 2008). This often allows for a significant reduction in the number of elements needed to represent complex 3D geometries. For example, a complex leaf shape can be represented by one or a few primitive elements rather than a triangular mesh consisting of dozens of elements (**Figure 2B**).

Upon the creation of each element, a minimal set of data is generated that defines the primitive, such as the coordinates of vertices, surface area, color, and so on. As is common in object-oriented programming, each element is assigned a unique universal identifier (UUID), which can be stored and later used to reference the element. This UUID can be passed to functions that, for example, apply a transformation to the element’s position, change an attribute of the element, or be passed to a model plug-in to indicate that model calculations should only be performed for that particular element. This allows for dynamic modification of geometry at any point within the program.

Primitive elements are the basis for most model data structures (**Figure 1**). Scalar or vector data of various types can be associated with each element called “primitive data” (e.g., temperature). These data can be used to specify unique model parameters for each element, or it can provide a container for values computed by a model for a particular primitive. These data structures are also how models are typically coupled. For example, one could create primitive data for each element that specifies its reflectivity, which would be read by the radiation model, which would then write another piece of primitive data that give the value of the computed radiation flux. Another model such as a photosynthesis model could then read this radiation flux and write additional primitive data that give the value of the computed photosynthetic flux. Primitive data values can be set or retrieved using the appropriate setter or getter function (see the section *C++ Application Programming Interface*), which normally takes the UUID of the associated element(s) as an argument.

There is also a more generic data container called “global data,” which are not associated with any single geometric element. Global data can be scalar or vector valued and can have a number of different data types (e.g., double, float, integer, string). Global data are set or retrieved using the appropriate setter or getter function, but do not require the UUID of a primitive element because they are independent of any single element.

The data structure formulation used in Helios allows for maximum flexibility in model coupling, but comes with the trade-off of decreased ease of use. The Context itself simply provides a flexible central repository for model geometry and associated data and can also handle file I/O if needed. For this reason, it is very general and allows for arbitrary model coupling and workflows. Plug-ins only need to know the name of the data objects it should read from and write to. Thus, plug-ins can be executed in any order and can share arbitrary data structures.

### Time-Series Data

Environmental models are commonly driven by time-series data provided by one or more sensors. The Helios Context includes tools to readily load and access these time-series data. Each data point is associated with some date and time and can either be read automatically from an XML file or added to the Context manually. By setting the date and time in the Context using the appropriate functions, the time-series data will be automatically interpolated to that instant in time and can be queried and used in model calculations.

### C++ Application Programming Interface

Users interact with Helios through a C++ application programming interface (API), which means that users write their own program that utilizes the Helios library (see **Listing 1**). As mentioned above, this offers high flexibility but decreases ease of use because users must write their own main function that declares and runs plug-ins. Many tutorials and examples are included within the Helios documentation that illustrate how to utilize the various data structures and functions to perform common modeling tasks.

The Helios Context is a C++ class with many public member functions that are used to access model geometry and data. Listing 1 provides example code for declaring the Context, adding a triangular element, and then setting primitive data for that element. In this example, the geometry is added through the Context member function “addTriangle(),” which takes the Cartesian coordinates of each triangle vertex as arguments. There are a number of additional overloaded versions of the addTriangle() function, which can be used to explicitly set the triangle color, set a texture map, and so on.

The API has several functions that can read/write from/to standard file formats, namely, XML, PLY, and OBJ formats. XML files are used to read and write simulation data and are based on a convention specific to Helios, which is detailed in the documentation. PLY (Stanford polygon) and OBJ (Wavefront) files are standard formats for storing geometric information and are read and written by most 3D computer graphics or computer-aided design software programs. This allows Helios to easily read 3D models generated by other software or write geometry created within Helios to formats that can be read by other software for further analysis. This enables a means by which geometry could be coupled or transferred between other plant modeling platforms that can handle these formats such as OpenAlea or GroIMP.

**Listing 1:** Example C++ code illustrating the procedure for using the Helios API to add geometry and set associated primitive data.

### API Documentation

Helios uses Doxygen (www.doxygen.org) to automatically generate documentation for the API and to create a user guide and tutorials with embedded hot-links to associated function documentation. Each plug-in has a documentation page with a consistent structure that defines several key aspects needed to work with the plug-in. This includes, but is not limited to, required dependencies, necessary header files, and any primitive or global data read or written by the plug-in. All API functions and data structures are searchable in order to quickly locate information regarding their purpose and function arguments.

## Plug-Ins

Helios plug-ins are implemented as C++ classes with a number of member functions that allow users to set up and run the models. The plug-in classes are typically passed a pointer to the Context class when they are declared, which gives them the ability to access data structures that define model geometry (e.g., vertex positions, surface area, normal vector) and read or write data structures in the Context. A brief description of plug-ins available in version 1.0 is given below, with corresponding software and hardware requirements given in **Table 1**.

**Table 1:** Summary of Helios plug-ins in version 1.0 and their respective software or hardware requirements.

Plug-in	Software/hardware requirements
Visualizer	X11/xorg packages
Radiation model	NVIDIA GPU, CUDA
Energy balance model	NVIDIA GPU, CUDA
Solar model	None
Stomatal conductance model	None
Photosynthesis model	None
Voxel intersection	NVIDIA GPU, CUDA
Procedural tree generation	None
LiDAR data processing	NVIDIA GPU, CUDA

### Visualizer

**Figure 3:**
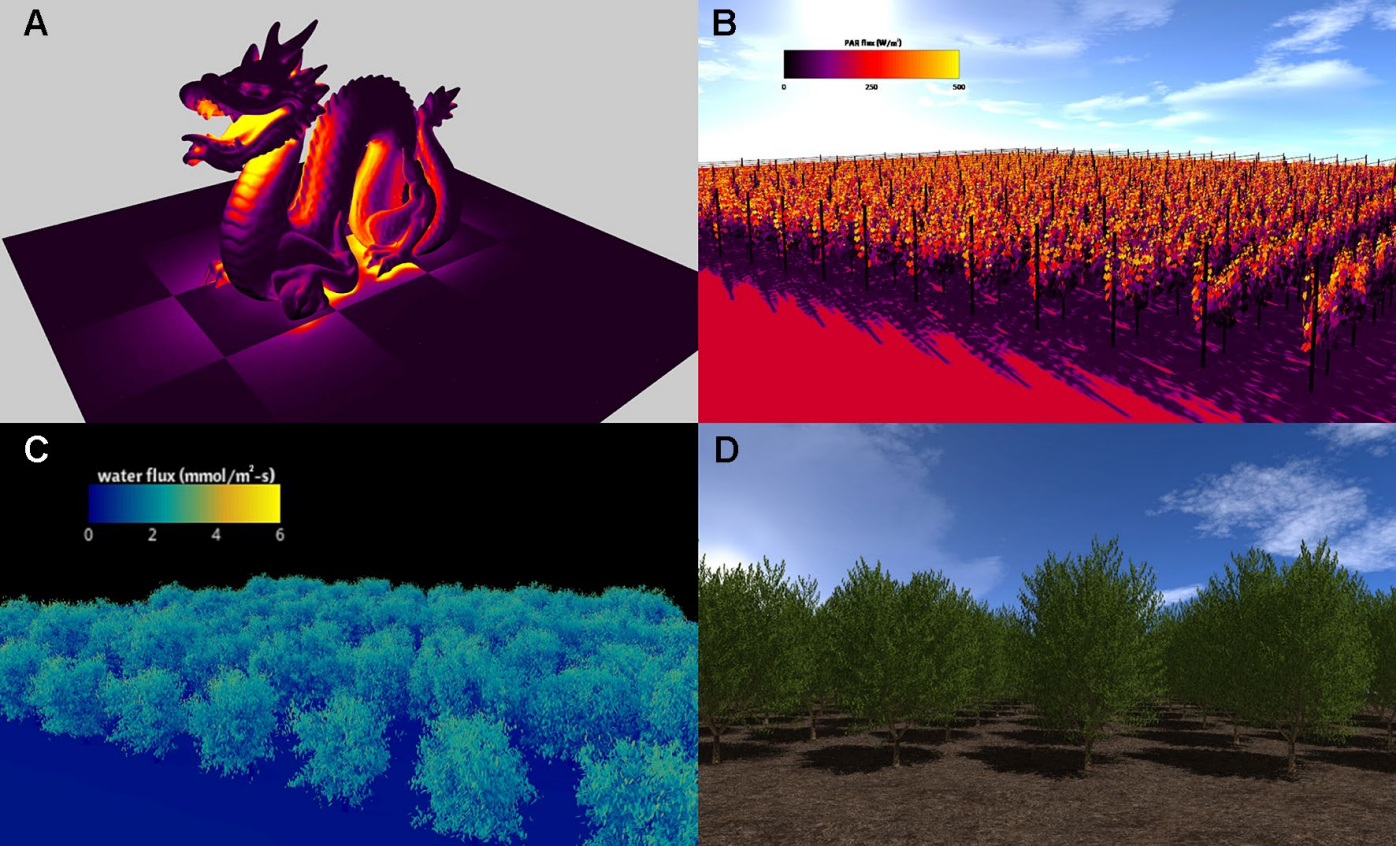
Visualizations of Helios simulation results. **(A)** simulation of radiation emission for model geometry read from a standard polygon file (the so-called “stanford dragon”); **(B)** simulation of absorbed photosynthetically active radiation in a vineyard; **(C)** simulation of transpiration flux in an almond orchard generated from terrestrial LiDAR reconstruction; **(D)** almond tree canopy geometry created using the procedural tree generation plug-in.

The visualizer plug-in creates 3D renderings of model geometry and data based on standard approaches used in computer graphics (Marschner and Shirley, 2015). Utilizing a pointer to the Context, the visualizer parses all geometric elements in the Context and renders them to the screen using OpenGL. There are several means by which elements may be colored. The user can specify a color for the element or provide the path to an image to be used for texture mapping (cf. Marschner and Shirley, 2015). In either case, the Phong lighting model can be optionally used to shade elements, with an additional option to use a model for shadow rendering (**Figure 3**). Alternatively, the user can specify that elements should be colored using a pseudocolor mapping based on primitive data stored in the Context (**Figure 3**).

While the visualizer plug-in provides a seamless means of quickly visualizing model outputs, it is also possible to output geometry and data to file using the standard formats introduced previously, which allows for the use of more sophisticated rendering tools such as Blender. The drawback of this approach is that it adds an additional step to the workflow.

### Radiation Transport Model

A GPU-accelerated model for radiation transfer is included as a plug-in to Helios, which is described in detail in Bailey (2018). The model uses a novel reverse ray-tracing approach for both solar radiation and terrestrial emission. While reverse ray-tracing approaches have been commonly used in previous models to provide more robust sampling of radiation sources (e.g., Lewis, 1999; Gastellu-Etchegorry et al., 2012; Henke and Buck-Sorlin, 2018), the model of Bailey (2018) presents a new reverse approach for modeling terrestrial emission that ensures that the model satisfies the second law of thermodynamics regardless of the number of rays used. The reduction in the number of rays required, along with the substantial acceleration achieved by utilizing a GPU-based parallelization, means that domains with hundreds of trees and tens of millions of fully resolved leaves can be simulated on a desktop workstation. Using simplified geometries with assumed radiative properties, Bailey (2018) showed that the model converges exponentially toward the exact analytical solution as the number of rays is increased. Currently, the model implementation does not support voxels, but a future release will include the ability to have a mixture of both planar primitive elements and voxels within the domain.

The model can be run over arbitrary wavebands, which are specified in the model by primitive data corresponding to surface radiative properties (i.e., absorptivity, reflectivity, emissivity) integrated over the particular waveband. External radiation sources can be represented by 1) a sphere, 2) collimated radiation propagating in a particular direction, or 3) diffuse ambient radiation, each of which also requires the specification of its position or direction as well as its emissive flux for each radiative band, which can be calculated using the solar model plug-in (see the section *Solar Position and Energy Model*).

### Surface Energy Balance Model

A surface energy balance can be solved for each primitive to calculate surface temperature and energy fluxes. The energy balance equation for a surface can be written as

(1) $$R-\varepsilon \sigma T_{s}^{4}=c_{p}g_{H}(T_{s}-T_{a})+\lambda g_{M}\left(\frac{e_{s}(T_{s})f_{s}-e_{s}(T_{a})h}{p_{atm}}\right)+Q_{other},$$

where *R* is the absorbed all-wave radiation flux, ε is the surface emissivity, σ = 5.67 × 10^–8^ W m^–2^ K^–4^ is the Stefan-Boltzmann constant, *T_s_* is the surface temperature in absolute units, *c_p_* = 29.25 J mol^−1^ K^−1^ is the heat capacity of air, *g_H_* is the surface boundary-layer conductance to heat, *T_a_* is the air temperature in absolute units, λ = 44,000 J mol^−1^ is the latent heat of vaporization of water, *g_M_* is the overall conductance to water vapor from the surface to air outside the surface boundary layer, *e_s_*(*T*) is the saturation vapor pressure at temperature *T* and is computed using the Tetens Equation (Campbell and Norman, 1998), *f_s_* is the fraction of water vapor saturation for the air immediately adjacent to the surface (by default *f_s_* = 1 for leaves assuming air in the substomatal cavity is saturated), *h* is the relative humidity of air outside the boundary layer, and *p_atm_* is the ambient air pressure. The flux *Q_other_* represents any additional energy fluxes that may be present at the primitive surface (e.g., storage). For the purposes of the case study presented below in the section *Case Study: Quantifying Leaf-Level Variability of Transpiration and Photosynthesis in Whole-Canopies*, it is noted that the rate of water loss *E* from the surface can be readily calculated from Eq. 1 by isolating the term *g_M_* (*e_s_*(*T_s_*)*f_s_* – *e_s_* (*T_a_*)*h*) / *p_atm_* = *E*.

All parameters in Eq. 1 can be either specified directly by the user, computed from another plug-in, or otherwise assumed to take the default value given in the documentation. The absorbed radiation flux *R* can be computed using the radiation transport model plug-in (Section 3.2), and in the case where the primitive corresponds to a leaf, the conductance to moisture *g_M_* can be computed using the stomatal conductance plug-in (see the section *Stomatal Conductance Model*). The energy balance equation is iteratively solved for each primitive in parallel on the GPU using the secant method (Press et al., 2007).

### Solar Position and Energy Model

A plug-in is available to estimate the position of the sun, as well as downwelling shortwave and longwave radiative fluxes. The solar position is calculated using standard astronomical relationships as described in Iqbal (2012). In the absence of direct measurements, the clear-sky solar radiative flux incident on a surface normal to the sun can be calculated using the REST2 model of Gueymard (2003). The REST2 model accounts for the effects of Rayleigh scattering and absorption due to water vapor, nitrogen dioxide, ozone, and aerosols. The model also provides an estimation of direct-diffuse partitioning of the incoming solar flux.

If measurements are not available, the downwelling longwave diffuse radiative flux can be calculated using this plug-in, which is based on the model of Prata (1996). The REST2 and longwave models both require specification of the precipitable water in the atmosphere, which is estimated using the model of Viswanadham (1981).

### Stomatal Conductance Model

Stomata are typically important active regulators of water vapor transport between the inside of leaves and the atmosphere (Jarvis and McNaughton, 1986). This regulatory effect is represented by specifying a stomatal conductance, which is modeled in Helios using the semimechanistic model of Buckley et al. (2012). This model represents stomatal conductance *g_s_* as a hyperbolic function of photosynthetically active photon flux density and local vapor pressure deficit, which is given by the equation

(2) $$g_{s}=\frac{E_{m}(Q+i_{0})}{k+bQ+(Q+i_{0})D},$$

where *Q* is the photosynthetically active photon flux density, and *D* is the vapor pressure deficit between the intercellular leaf air spaces and the air outside of the leaf boundary layer. Note that the photon flux density is obtained from the energy flux in the PAR band using the factor 4.57 μmol m^−2^ s^−1^/(W m^−2^). *E_m_*, *i_0_*, *k*, and *b* are treated as empirical model coefficients.

### Photosynthesis Model

Two leaf photosynthesis models are available in Helios: an empirical model based on the description of Johnson (2010), and the mechanistic biochemical model of Farquhar et al. (1980) for C3 photosynthesis. For completeness, the current implementation of the Farquhar et al. (1980) model is described in Appendix 1 because it is the model used in the case study presented in the section *Case Study: Quantifying Leaf-Level Variability of Transpiration and Photosynthesis in Whole-Canopies*. The empirical model is also fully described in the Helios documentation.

### Primitive Subvolume Grouping

One important motivation for using a detailed, leaf-resolving plant model is to understand the impacts of aggregation of leaf-level heterogeneity over multiple scales. In order to help facilitate this aggregation, a plug-in is available to rapidly group or bin primitives into arbitrary subvolumes. Users can define arbitrary voxels, and this plug-in will identify any planar primitive elements that are contained within each voxel. This is useful, for example, in calculating leaf area density/index or calculating aggregated attenuation coefficients for comparison with simple models. The primitive binning calculations are performed on the GPU to significantly reduce execution times.

### Terrestrial LiDAR Data Processing

Terrestrial LiDAR scanning is a powerful tool for 3D measurement of plant architecture, which has gained popularity in plant modeling applications. While the raw LiDAR point clouds provide a wealth of data that yield an incredibly detailed mapping of the canopy, processing this data into information that is usable in the context of modeling has proven to be a challenge. Raw LiDAR data provide millions of 3D Cartesian coordinates in space. However, models generally cannot use points directly, but rather need information such as surfaces, areas, and so on.

The terrestrial LiDAR plug-in integrates a number of data processing algorithms, along with GPU acceleration, to provide the ability to translate LiDAR point clouds into leaf-by-leaf reconstructions that can be fed directly into the Helios Context. The workflow starts by using the triangulation algorithm of Bailey and Mahaffee (2017b) to calculate the leaf angle distribution, which is used to calculate the leaf area projection function *G* (Ross, 1981). The G-function is then used to generate estimates of leaf area density for arbitrary volumes of leaves (voxels) following the approach of Bailey and Mahaffee (2017a). To reconstruct individual leaves, the triangulated leaf hit points are segmented to estimate the position and area of individual leaves that are in direct view of the LiDAR scanner (Bailey and Ochoa, 2018). Because a significant fraction of leaves may be occluded from view of the scanner, a statistical backfilling approach is used to ensure that the reconstructed tree leaf orientation and area distributions match the voxel-based measurements described above (see Bailey and Ochoa, 2018).

Each individual LiDAR scan typically consists of tens of millions of points, and grids for calculating leaf area density may consist of thousands of voxels. These dimensions compound to make data processing computationally expensive, and thus several of the LiDAR processing routines are performed in parallel on the GPU. Point-based calculations lend themselves well to parallelization because each laser pulse can be analyzed independently from another.

### Procedural Tree Generation

While the LiDAR plug-in provides a powerful means of incorporating measured tree architectures within Helios, certain types of modeling studies may require the ability to simulate a wide range of geometries that cannot be directly measured. The creation of semirandom tree geometries is made possible in Helios through the use of the procedural tree generation algorithm of Weber and Penn (1995). This framework describes the woody architecture of trees as a recursive set of branching levels, each described by their own set of parameters that provide rules for how branching structure should occur. A random perturbation of user-defined magnitude can be added to each parameter to reduce geometric uniformity in order to produce more realistic-looking trees. In the original formulation described by Weber and Penn (1995), leaf orientations are determined through an axial rotation about the branch from which they originate, which may create unphysical leaf orientation distributions. Additional functionality has been added to allow users to specify a custom leaf inclination angle distribution, perhaps that provided by LiDAR measurements (section *Terrestrial LiDAR Data Processing*).

**Figure 4:**
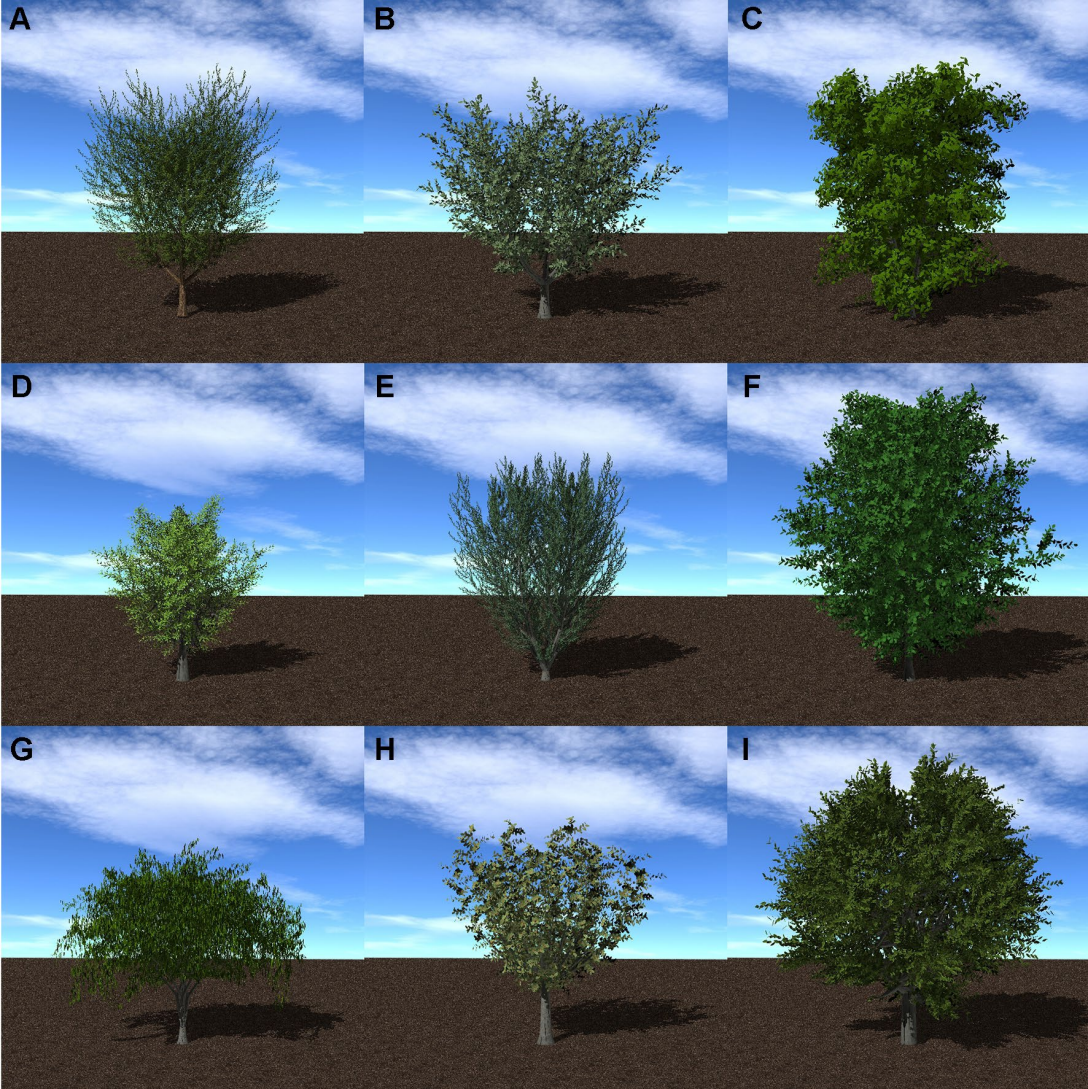
Procedural tree model generation for nine tree species: **(A)** almond; **(B)** apple; **(C)** avocado; **(D)** lemon; **(E)** olive; **(F)** orange; **(G)** peach; **(H)** pistachio; **(I)** walnut.

The procedural tree generation plug-in comes with nine predefined tree geometries, which are shown in **Figure 4**. Arbitrary trees can be created by modifying the tree geometric parameters, which are commonly specified in an XML file. Parameters include quantities such as the number of recursive branching levels, average angle of branches with respect to their parent branch for each level, and so on. The end geometry produced by the Weber and Penn (1995) and the parameters used to specify the geometry are fairly similar to those produced by the commonly used L-systems approach (Prusinkiewicz and Runions, 2012). L-systems is more elegant in its notational and mathematical representation of the branching structures (it uses a string of characters to encode the structure), but the end result is similar to that used in Helios.

## Case Study: Quantifying Leaf-Level Variability of Transpiration and Photosynthesis in Whole-Canopies

### Background

While our collective understanding of plant biophysical processes for individual leaves has progressed rapidly over the past several decades, our understanding of canopy-level processes is limited by the need to aggregate highly heterogeneous processes over a wide range of scales. When measurements are performed at the leaf scale, it is often unclear how representative such measurements are of the canopy as a whole. On the other hand, when measurements are performed at large scales that aggregate many smaller scales, it is often unclear how different members of the community (i.e., leaves) contribute to aggregate behavior. In this brief case study, a tree canopy will be examined using Helios to visualize and quantify leaf-level variability in transpiration and photosynthesis in order to understand how individual elements contribute to system-level behavior.

### Case Set-up

Canopies of *Prunus dulcis* were simulated to assess the impact of canopy architecture on light interception, microclimate, transpiration, and photosynthesis. Two canopy architectures were considered: an isolated tree and a relatively dense canopy of 100 trees (1 tree per 36 m^2^). Individual trees were created using the procedural tree generation plug-in with the same parameters that were used to create the tree shown in **Figure 4A**. In order to maintain consistency within the test case, the isolated tree had the same architecture (within random variation) of each of the trees in the dense canopy, although in reality the architecture of the isolated tree would likely be different. The simulated trees had leaves with constant (one-sided) area of 60 cm^2^.

**Figure 5:**
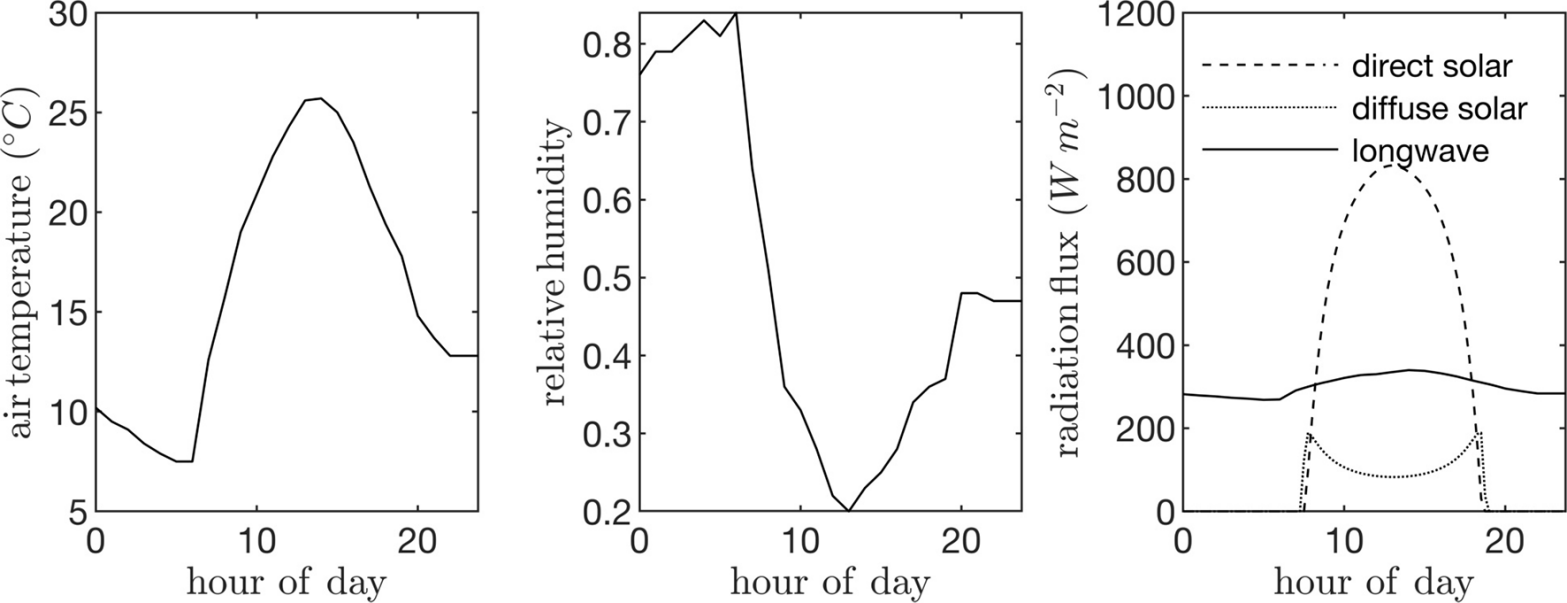
Input time-series data used to drive case study simulations: ambient air temperature (left), air relative humidity (center), and incoming direct solar radiation flux normal to the sum direction, incoming diffuse radiation flux on a horizontal surface, and incoming longwave radiation flux on a horizontal surface (right).

Field data collected in a canopy of *P. dulcis* were used to specify model parameters. The canopy was located in the California Central Valley (36.599°N 119.515°W), and consisted of 4-year-old trees that were approximately 7 m tall. Trees were spaced at 4 m in the East-West direction and 6.4 m in the North-South direction. The ambient air temperature and humidity were assumed to be spatially homogeneous and were specified using data collected from a nearby weather station (**Figure 5**). These data were also fed into the model that predicts the downwelling longwave radiation flux. Incoming direct and diffuse radiation fluxes were estimated using the REST2 model as described above (**Figure 5**), which were equally partitioned into PAR (assumed to be wavelengths <700 nm) and NIR (assumed to be wavelengths >700 nm) bands. A single diurnal cycle was simulated at time step of 15 min. Leaf reflectivity and transmissivity were assumed to be 0.05 for the PAR band and 0.4 for the NIR band.

**Figure 6:**
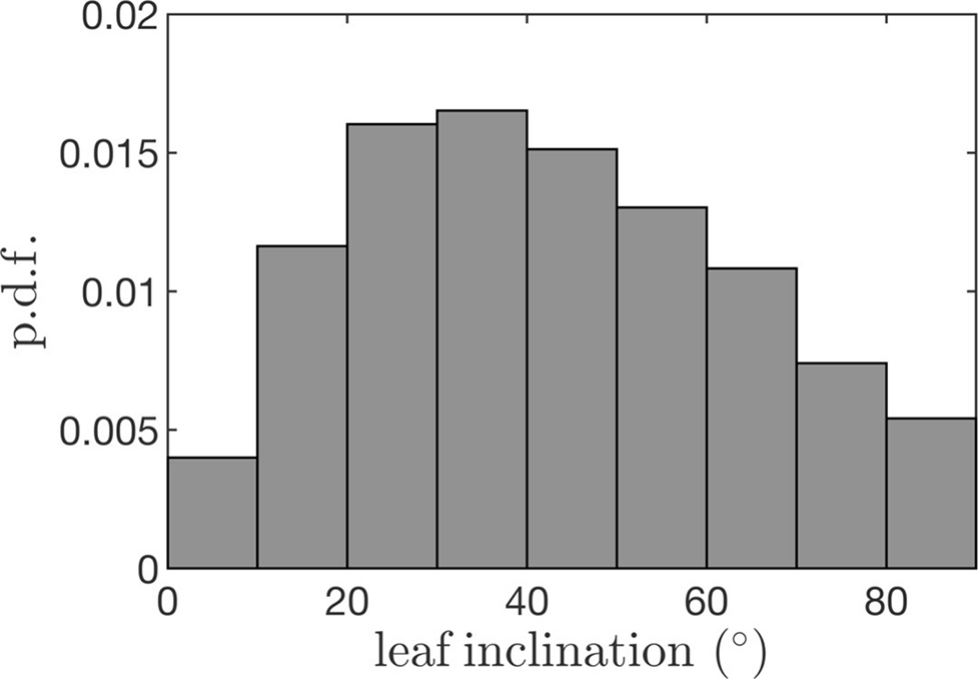
Leaf inclination probability density function (p.d.f.) used to generate tree geometrics for case study simulations.

Leaf angles were specified by randomly drawing from the leaf angle distribution measured in the experimental canopy described above. The average leaf angle distribution was measured by scanning trees using a Riegl VZ-1000 terrestrial LiDAR scanner (Horn, Austria). The scan resolution in the zenithal direction was 0.04° across a range of 100° and 0.08° in the azimuth across a full 360° rotation. Four scans per tree were performed from the northwest, northeast, southwest, and southeast of each trunk at a distance of about 7.5 m. The raw LiDAR data were processed to determine the leaf inclination distribution as described by Bailey and Mahaffee (2017b) using the LiDAR data processing plug-in (**Figure 6**).

**Figure 7:**
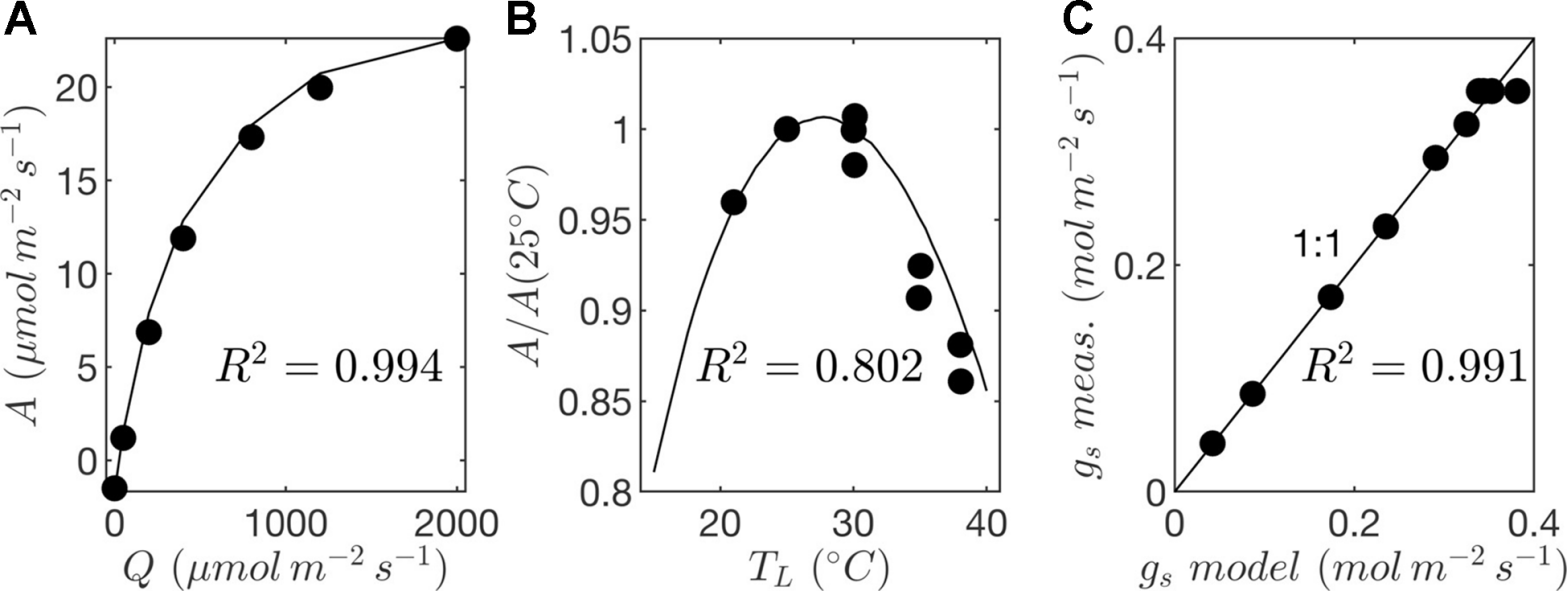
Parameterization of photosynthesis and stomatal conductance models. **(A)** response of net photosynthesis to varying photon flux density (*Q*) at a reference leaf temperature of *T_L_* = 25ºC given by gas exchange measurements (circles) and photosynthesis model (solid line); **(B)** response of net photosynthesis to temperature (*T_L_*) given by gas exchange measurements (circles) and photosynthesis model (solid line); and **(C)** comparison of stomatal conductance (*g*_s_) given by gas exchange measurements and predictions by the model.

Leaf-level gas exchange measurements were collected using the LI-6800 portable photosynthesis system (LICOR, Lincoln, NE, USA). All measurements were performed at an ambient CO2 concentration of 390 μmol/mol, but at varying light, temperature, and humidity levels. The response of photosynthesis and stomatal conductance to light was determined by varying the photosynthetically active photon flux density between levels of 0, 50, 200, 400, 800, 1,200, and 2,000 μmol m^−2^ s^−1^ at a leaf temperature of 25°C and 60% relative humidity. Importantly, a large amount of time was spent at each light level to ensure that stomata had time to fully equilibrate, which took around 1 h per light level. Control measurements with constant conditions were performed to verify that changes in whole-plant water status over this very long period did not significantly affect the response curves. At a saturating light level of 2,000 μmol m^−2^ s^−1^, leaf temperature varied between 25°C and 35°C, and relative humidity in the chamber varied between 30% and 60% for each leaf temperature. This procedure produced measurements at 10 different combinations of light, temperature, and ambient humidity, which were used to determine model coefficients for the stomatal conductance and photosynthesis models (**Figure 7**; **Table 2**).

**Table 2:** Fitted parameter values for photosynthesis and stomatal conductance models.

Parameter	Description	Value	Units
		*Photosynthesis*	
*R_d,25_*	Respiration rate at 25ºC	1.491	*µ*mol m^−2^ s^−1^
*V_cmax,25_*	Maximum carboxylation rate at 25ºC	99.5	*µ*mol m^−2^ s^−1^
*J_max,25_*	Maximum electron transport rate at 25ºC	185.0	*µ*mol m^−2^ s^−1^
*C_Jmax_*	*J_max_* temperature response parameter	17.57*	Unitless
*ΔH_a, Jmax_*	*J_max_*temperature response parameter	43.54*	kJ mol^−1^
*α*	Light response parameter	0.41	Unitless
		*Stomatal Conductance*	
*E_m_*	Maximum transpiration rate	20.43	mmol m^−2^ s^−1^
*i_0_*	PPFD offset for dark transpiration	38.48	*µ*mol m^−2^ s^−1^
*k*	Bulk stomatal parameter	18,383	*µ*mol m^−2^ s^−1^ mmol mol^−1^
*b*	Bulk stomatal parameter	49.68	mmol mol^−1^

*Assumed based on no growth temperature acclimation ( Bernacchi et al., 2003 ).

In order to refine the initial representation of canopy architecture, a precursor simulation was performed to remove unrealistic leaves. The daily net CO2 assimilation rate was determined for every leaf within the precursor simulation. Leaves that had negative net CO2 assimilation over the day (i.e., daily respiration was larger than assimilation) were removed. This resulted in a final canopy LAI of 2.9, which had very few leaves with negative net daily CO2 assimilation (**Figure 9**). This LAI value is on the high end of what might be observed in real canopies but is reasonable given that nearly all leaves had positive net daily assimilation.

### Results

#### Leaf Probability Distributions

**Figure 8:**
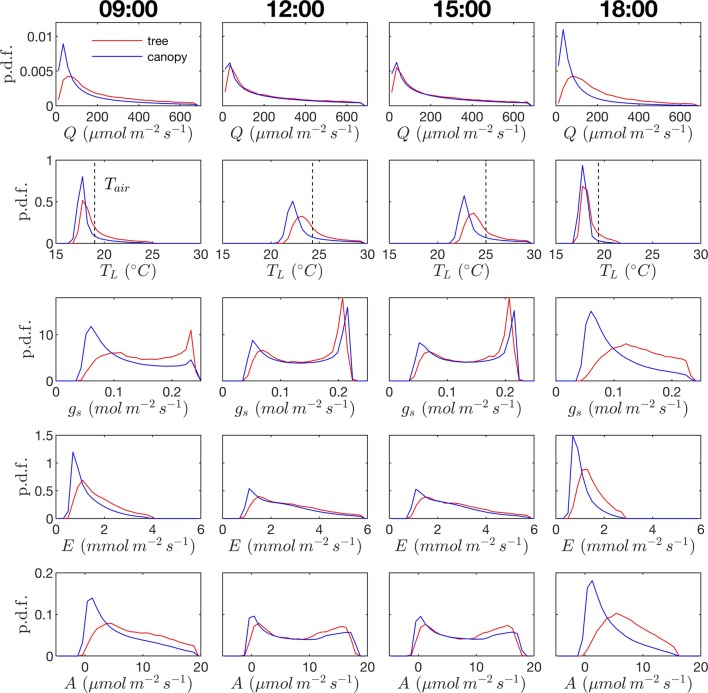
Probability density function (p.d.f.) across all leaves in the canopy for photosynthetic photon flux density *Q*, leaf temperature *T_L_* (ambient temperature given by dashed vertical line), stomatal conductance to water vapor *g*_s_, transpiration rate *E*, and net rate of photosynthesis A. Columns correspond to different times of the day. Red lines correspond to the isolated tree case; blue lines correspond to the dense canopy case

**Figure 9:**
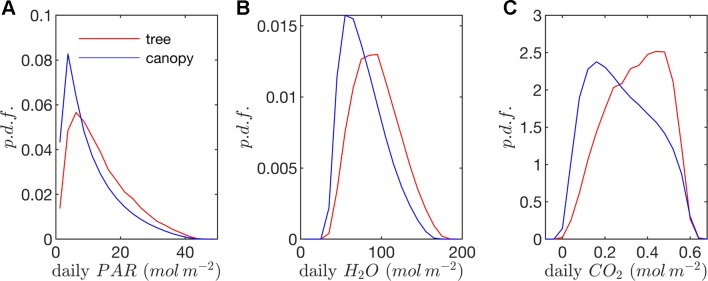
Probability density functions (p.d.f.s) of daily **(A)** absorbed photosynthetic photons, **(B)** transpired water, and **(C)** net CO_2_ exchange (all per unit leaf area).

Probability distribution functions (p.d.f.s) of net photosynthesis, transpiration rate, absorbed radiation, and temperature were calculated for all leaves in the tree or canopy. The distributions were formed across leaves for given instants throughout the day (**Figure 8**) or as an integration in time of values for each leaf over the entire day for daylight hours only (**Figure 9**).

**Figure 10:**
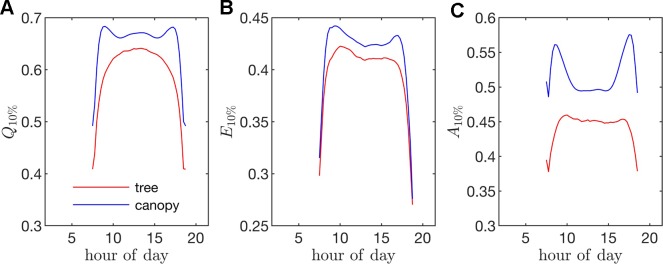
Fraction of the instantaneous flux due the top 10% of all leaves (i.e., fraction of flux contained in the 90^th^ percentile of leaves) for fluxes of **(A)** absorbed photon flux *Q*_10%_, **(B)** transpiration rate *E*_10%_, and **(C)** net photosynthesis *A*_10%_. (For example, a *Q*_10%_ value of 0.75 would mean that only 10% of the leaves were responsible for 75% of the total absorbed PAR for the whole tree/canopy.

*Radiation flux*. The distribution of absorbed radiation was highly heterogeneous, and followed a nearly exponential distribution, with most leaves absorbing relatively low amounts of radiation. This exponential distribution was an expected result based on Beer’s law (Ross, 1981), as the p.d.f. of absorbed flux over all leaves serves to approximate the probability of flux interception along the path of radiation propagation. The distribution is not perfectly exponential due to the presence of diffuse ambient radiation and the fact that the tree/canopy is not optically thick, and therefore the ground absorbs some radiation. The instantaneous and daily integrated p.d.f.s showed a similar trend, except that the daily p.d.f. had a shorter tail. The highly skewed distribution meant that a relatively small number of leaves absorbed a large fraction of radiation at any instant of the day. Around the middle portion of the day, leaves in the top 10% in terms of absorbed radiation flux absorbed roughly 65% of the total radiation absorbed by the entire tree or canopy (**Figure 10A**). As the sun angle decreased, this fraction tended to decline, where near dawn and dusk the top 10% of leaves absorbed between 40% and 50% of the total absorbed radiation (**Figure 10A**), which is likely due to the increased diffuse fraction. When integrated over the entire day, 10% of leaves were responsible for absorbing about 48% of the total daily absorbed radiation for the isolated tree and 53% for the dense canopy (**Table 3**).

**Table 3:** Fraction of the total daily flux due the top 10% of all leaves (i.e., fraction of total flux contained in the 90th percentile of leaves) for fluxes of absorbed photon flux *Q*_10%_, transpiration rate *E*_10%_, and net photosynthesis *A*_10%_.

Variable	Tree	Canopy
*Q*_10%_	0.480	0.533
*E*_10%_	0.351	0.367
*A*_10%_	0.370	0.432

The probability distributions of absorbed radiation for the isolated tree and dense canopy cases were very similar when the sun was high, and decreasing sun angle tended to smooth the distribution slightly for the isolated tree (**Figure 8**). The distribution for the isolated tree was shifted slightly to higher radiation values, likely due to relatively high fraction of surface area in view of the sun at low sun angles. When integrated over an entire day, the discrepancies between the distributions for the isolated tree and dense canopy were relatively minimal, with the peak in the distribution smoothed slightly for the isolated tree (**Figure 9A**).

*Leaf temperature*. The distributions of leaf temperature were closer to Gaussian than the distributions of radiation absorption, although the temperature distributions were still positively skewed (**Figure 8**). Most leaves were below the ambient air temperature, with the peak occurring several degrees below the ambient air temperature. There was a significant difference between the leaf temperature distributions for the isolated tree and dense canopy cases, particularly throughout the middle portion of the day. The lower end of the leaf temperature distributions for the tree was shifted upward by about a degree when compared with the dense canopy. The upper end of the leaf temperature distributions is similar between the tree and canopy cases, indicating that the highest temperature leaves, likely near the tops of the trees, are not significantly affected by the presence of neighboring trees. However, it should be noted that this includes only the radiative effect on temperature because the air temperature was held constant between the isolated tree and canopy cases in order to isolate the effects of geometry.

*Stomatal conductance*. During the middle portion of the day, the distribution of leaf stomatal conductance followed an interesting bimodal distribution with sharp peaks at either end of the distribution, which also exhibited minimal differences between the isolated tree and dense canopy cases. The lower peak results from the fact that a large portion of leaves are in shade, resulting in a large number of leaves with low stomatal conductance. The upper peak is perhaps more surprising and results from the nonlinearity of the stomatal response to light. For high light levels, stomatal conductance saturates and is relatively insensitive to changes in light, which thus results in a large cluster of leaves with stomatal conductances near the saturating value. Late in the day when sun angles are low, there is a significant positive shift in stomatal conductances in the isolated tree as compared with the dense canopy, which seemingly corresponds with the positive shift in radiation absorption between these two cases.

*Transpiration rate*. Unlike the distribution of stomatal conductance, the distribution of transpiration flux did not follow a bimodal distribution, but rather had a single sharp peak and large positive skewness. Since the transpiration flux is the product of the stomatal conductance and vapor pressure deficit, this means that the vapor pressure deficit increase at high temperature and light values was sharp enough to dominate the transpiration flux, although stomatal conductance becomes saturated. Overall, discrepancies between the highest and lowest transpiring leaves were smaller than those of absorbed radiation. During much of the day, the top 10% of leaves transpired between 40% and 45% of the total tree/canopy transpiration (**Figure 10B**), and when integrated over the day, the top 10% transpired roughly 35% of the total for both the tree and canopy cases.

The peak in the distribution of transpiration flux was shifted upward in the isolated tree case, which was presumably due to the corresponding upward shift in the leaf temperature and stomatal conductance (**Figure 8**). When the transpiration flux was integrated over the entire day, a similar pattern emerged, except that the positive tail of the distribution was shortened (**Figure 9**).

*Net photosynthesis*. During the middle portion of the day, the distribution of net leaf CO2 flux exhibits a peak near a value of zero and a secondary peak near the saturating value (**Figure 8**), with the overall distribution being fairly uniform. At low light levels, photosynthesis is primarily limited by the amount of available photosynthetically active light, and thus it is expected that for low light values the distribution of photosynthesis should be closely related to the distribution of absorbed light, which is evident from **Figure 8**. At high light levels, photosynthesis is relatively insensitive to light (**Figure 7**) but highly sensitive to the CO2 concentration within the leaf, which is tightly regulated by stomatal conductance. When integrated over an entire day, a strong peak in the photosynthesis distribution at low CO2 exchange values still exists but is shifted upward, and the region of nearly constant CO2 exchange values does not exist (**Figure 9**). For the middle of the day, the top 10% of leaves assimilated about 45% of the CO2 for the isolated tree and 50% for the canopy (**Figure 10C**). When integrated over the day, the top 10% of leaves assimilated 37% of the CO2 in the isolated tree and 43% in the dense canopy.

### Leaf Trajectories

**Figure 11:**
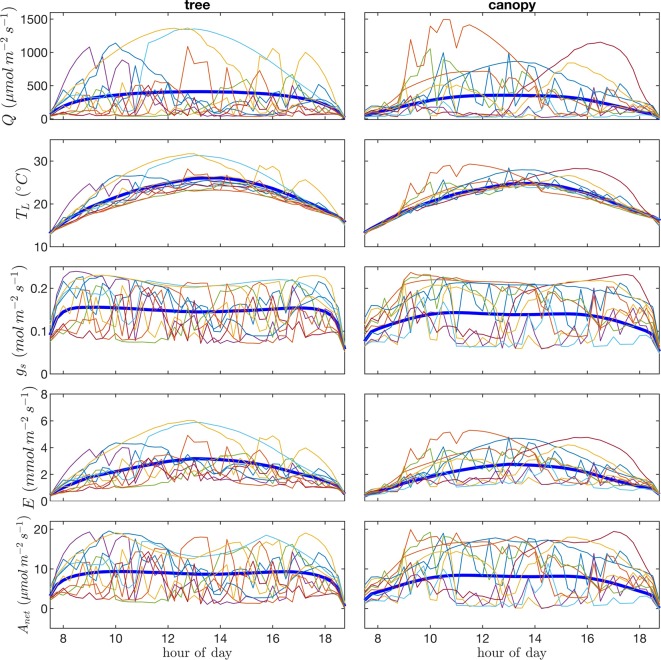
Time-series or “trajectories” for 10 randomly selected leaves of photosynthetic photon flux density *Q*, leaf temperature *T_L_*, stomatal conductance *g*_s_, transpiration flux *E*, and net photosynthetic flux *A*. Each line/color represents the time series of a single leaf. Thick blue lines give the average over all leaves in the tree canopy.

Visualization of time series or “trajectories” of individual leaf exchange rates provides an interesting perspective into how the behavior of individual leaves compares to that of the entire tree or canopy throughout the day. Trajectories are shown in **Figure 11** for absorbed photosynthetically active radiation, leaf temperature, stomatal conductance, transpiration rate, and net rate of photosynthesis for 10 randomly chosen leaves. We can observe the wide range of scenarios encountered by different leaves. Some leaves remain in highly shaded conditions for most of the day except for a brief sunfleck, which allows them to assimilate enough CO2 to offset daily respiration. Other leaves are “lucky” in that they encounter extended periods of high light conditions. Examples can be observed in which the leaf radiation, temperature, and transpiration rate all increase substantially and in tandem for an extended period, whereas stomatal conductance and photosynthesis reach a maximum value and begin to decline as vapor pressure deficit climbs and stomata start to close.

## Discussion

The goal of most modeling efforts is to reduce complex processes to a tractable form that can mathematically represent interrelationships between quantities of interest. Here, our goal was to use a complex model that represents in detail individual members of a complex system (i.e., leaves in a tree/canopy) to help identify emergent behavior that is largely representative of the bulk response of the system, which can provide insight into how simplified experimental and modeling approaches can be formulated and interpreted. In this brief case study, Helios and its submodels for radiation transport, leaf temperature, stomatal conductance, and photosynthesis were used to examine leaf-level variability in these processes and how this variability contributes to whole-tree and -canopy behavior.

The results of this case study provide an interesting depiction of the extreme heterogeneity that exists within vegetation for important biophysical processes. Probability distributions across leaves are highly heterogeneous and skewed, and because of inherent nonlinearities in the biophysical processes examined, the general shape of distributions is not consistent across even tightly related processes. At any instance, the whole-tree/canopy behavior in terms of radiation interception and photosynthesis is dominated by a relatively small fraction of the leaf population. When integrated over an entire day, this effect is somewhat reduced, but it was still observed that a small fraction of leaves was responsible for a disproportionate amount of the daily CO2 assimilation.

A wide range of representations of the above biophysical processes are used in models. So-called “big-leaf” models consider the behavior of only one average leaf assumed to be representative of the entire plant system (e.g., Sinclair et al., 1976; Sellers et al., 1986; Amthor, 1994). For the tree systems examined here, **Figures 8** and **9** illustrate the difficulties in utilizing this approach, given the high variability and skewness of the distributions across leaves, which has also been highlighted in more recent works (De Pury and Farquhar, 1997; Wang and Leuning, 1998; Friend, 2001). As an improved, yet still simple approximation, authors have suggested choosing two representative sets of leaves: sunlit and shaded (De Pury and Farquhar, 1997; Wang and Leuning, 1998). Examination of the distributions of absorbed radiation in **Figures 8** and **9** would call this intuitive approximation into question. Although the naked eye may view two distinct radiation regimes within a tree, this can be deceiving given that leaves are at a variety of orientations with respect to incoming radiation. No clear separation of regimes is evident for absorbed radiation, temperature, and transpiration, although stomatal conductance and photosynthesis had two distinct peaks in the distribution for the middle portion of the day. The degree of separation between sunlit and shaded regimes is expected to vary based on the shape of the light response curve (**Figure 7A**) and the density of vegetation.

More complicated “multilayer models” (e.g., Meyers and Paw U, 1987; Bonan et al., 2012) appear suitable for representing the within-vegetation heterogeneity, provided that enough vertical layers are used. Subdividing the canopy into discrete zones effectively averages across all values within the zone. It is possible that using a zone that is too large can introduce problems due to the fact that the distribution within that zone can have fat tails that give large contributions to overall behavior.

The comparisons between the isolated tree and dense canopy in this study showed surprisingly small differences in the distributions of radiation absorption, transpiration, and photosynthesis through most of the day and in daily integrated distributions, which raises some interesting questions regarding the representation of isolated or sparse vegetation in simplified biophysical models. Because both the isolated tree and canopy cases showed a nearly identical exponential distribution in absorbed radiation, a simple homogeneous Beer’s law model could conceivably be used to predict total absorbed radiation per unit total leaf area for the isolated tree. However, the complication arises that we must know the total leaf area and representative ground area for the isolated tree to get an absorbed flux per unit ground area. Models that aggregate trees into homogeneous subvolumes (e.g., see Wang and Jarvis, 1990; Cescatti, 1997; Duursma and Medlyn, 2012) correctly represent tree-scale heterogeneity in absorption, but filter out subtree variability including the tails of the distributions, which were shown to have important contributions to whole-canopy behavior. On the other hand, multilayer models can represent this subtree variability but are not able to represent tree-level heterogeneity in sparse canopies (Ponce de Len and Bailey, 2019).

## Data Availability Statement

Helios is an open-source software licensed under the GNU GPLv3 license. It can be downloaded from the GitHub repository located at https://github.com/PlantSimulationLab/Helios.

## Author Contributions

The author confirms being the sole contributor of this work and has approved it for publication.

## Funding

Financial support of this work by the American Vineyard Foundation grants 2015-1825/2016-1825/2017-1825, Almond Board of California grants 17.PREC1/18.PREC1, and the USDA National Institute of Food and Agriculture Hatch project 1013396.

## Conflict of Interest

The author declares that the research was conducted in the absence of any commercial or financial relationships that could be construed as a potential conflict of interest.

## Appendix 1 Photosynthesis Model Equations

Diffusion of CO2 into and out of the leaf is modeled using the following resistance analogy:

(3) $$A+R_{d}=0.75g_{M}(C_{a}-C_{i}),$$

where is the CO2 assimilation rate, *R_d_* is the respiration rate, and 0.75*g_M_* is the conductance to CO2 transport between the mesophyll and ambient air, which is assumed to differ from water vapor diffusion only based on its lower diffusivity in air (and hence the 0.75 factor). Diffusion is driven by a difference between the intercellular CO2 concentration *C_i_* and the air CO2 concentration *C_a_*.

The CO2 assimilation rate is calculated following Farquhar et al. (1980) as

(4) $$A=\left(1-\frac{\Gamma *}{C_{i}}\right)\min\{W_{c},W_{j}\}-R_{d},$$

where Γ*** is the chloroplastic CO2 compensation point. *A* is limited either by the Rubisco-limited carboxylation rate *W_c_*, or by the rate of RuBP regeneration *W_j_* (we neglect the TPU limitation state for conditions typical of the natural environment). *W_c_* is calculated according to

(5) $$W_{c}=\frac{V_{\mathrm{cmax}}C_{i}}{C_{i}+K_{c}(1+O/K_{O})},$$

where *V_c_*,*_max_* is the maximum carboxylation rate, *K_c_* is the Michaelis-Menten constant for RuBP carboxylation, *K_o_* is the Michaelis-Menten constant for oxygenation, and *O* is the partial pressure of oxygen in the air. *W_j_* is calculated according to

(6) $$W_{c}=\frac{JC_{i}}{4C_{i}+8\Gamma *},$$

where the potential electron transport rate *J* is modeled using the hyperbolic relationship

(7) $$J=\frac{J_{\max}Q\alpha }{Q\alpha +J_{\max}},$$

where *J_max_* is the value of *J* at saturating *Q*, and α describes the rate at which *J* reaches *J_max_* with increasing *Q*.

In addition to the original formulation proposed by Farquhar et al. (1980), the temperature dependence of model parameters has also been included following the description given by Bernacchi et al. (2001) and Bernacchi et al. (2003), which are given by the following equations:

(8a) $$\Gamma^{*}=\text{exp}(19.02-37.83/(RT_{L})),$$

(8b) $$K_{c}=\text{exp}(38.05-79.43/(RT_{L})),$$

(8c) $$K_{O}=\text{exp}(20.30-36.38/(RT_{L})),$$

(8d) $$R_{d}=R_{d,25}\exp(18.72-46.39/(RT_{L})),$$

(8e) $$V_{cmax}=V_{cmax,25}\exp(26.35-65.33/(RT_{L})),$$

(8f) $$J_{cmax}=J_{max,25}\exp(C_{Jmax}-∆H_{a,Jmax}/(RT_{L})),$$

where *R* is the universal gas constant, *T_L_* is the leaf temperature in absolute units, and the subscript 25 indicates the evaluation of the parameter at a temperature of 25°C. This leaves the following free parameters to be specified in the photosynthesis model: *R_d,25_*, *V_cmax_*_,25_, *J_max_*_,25_, α, *C_Jmax_*, and Δ*H_a_*,*_Jmax_*.

## References

[B1] Allen M. T. Prusinkiewicz P. DeJong T. M. (2005). Using L-systems for modeling source-sink interaction, architecture and physiology of growing trees: the L-PEACH model. New Phytol. 166, 869–880. 10.1111/j.1469-8137.2005.01348.x 15869648

[B2] Amthor J. S. (1994). Scaling CO_2_-photosynthesis relationships from the leaf to the canopy. Photosyn. Res. 39, 321–350. 10.1007/BF00014590 24311128

[B3] Bailey B. N. (2018). A reverse ray-tracing method for modelling the net radiative flux in leaf-resolving plant canopy simulations. Ecol. Model. 398, 233–245. 10.1016/j.ecolmodel.2017.11.022

[B4] Bailey B. N. Mahaffee W. F. (2017a). Rapid, high-resolution measurement of leaf area and leaf orientation using terrestrial LiDAR scanning data. Meas. Sci. Technol. 28, 064006. 10.1088/1361-6501/aa5cfd

[B5] Bailey B. N. Mahaffee W. F. (2017b). Rapid measurement of the three-dimensional distribution of leaf orientation and the leaf angle probability density function using terrestrial LiDAR scanning. Remote Sens. Environ. 193, 63–76. 10.1016/j.rse.2017.03.011

[B6] Bailey B. N. Ochoa M. H. (2018). Semi-direct tree reconstruction using terrestrial LiDAR point cloud data. Remote Sens. Environ. 208, 133–144. 10.1016/j.rse.2018.02.013

[B7] Bailey B. N. Overby M. Willemsen P. Pardyjak E. R. Mahaffee W. F. Stoll R. (2014). A scalable plant-resolving radiative transfer model based on optimized GPU ray tracing. Agric. For. Meteorol. 198-199, 192–208. 10.1016/j.agrformet.2014.08.012

[B8] Bailey B. N. Stoll R. Pardyjak E. R. Miller N. E. (2016). A new three-dimensional energy balance model for complex plant canopy geometries: model development and improved validation strategies. Agric. For. Meteorol. 218-219, 146–160. 10.1016/j.agrformet.2015.11.021

[B9] Baldocchi D. D. Harley P. C. (1995). Scaling carbon dioxide and water vapour exchange from leaf to canopy in a deciduous forest. II. model testing and application. Plant Cell Environ. 18, 1157–1173. 10.1111/j.1365-3040.1995.tb00626.x

[B10] Bernacchi C. J. Pimentel C. Long S. P. (2003). *In vivo* temperature response functions of parameters required to model RuBP-limited photosynthesis. Plant Cell Environ. 26, 1419–1430. 10.1046/j.0016-8025.2003.01050.x

[B11] Bernacchi C. J. Singsaas E. L. Pimentel C. JR A. R. P. Long S. P. (2001). Improved temperature response functions for models of rubisco-limited photosynthesis. Plant Cell Environ. 24, 253–259. 10.1111/j.1365-3040.2001.00668.x

[B12] Bonan G. B. Oleson K. W. Fisher R. A. Lasslop G. Reichstein M. (2012). Reconciling leaf physiological traits and canopy flux data: use of the TRY and FLUXNET databases in the Community Land Model version 4. J. Geophys. Res. Biogeosci. 117, G02026. 10.1029/2011JG001913

[B13] Boudon F. Pradal C. Cokelaer T. Prusinkiewicz P. Godin C. (2012). L-Py: an L-system simulation framework for modeling plant architecture development based on a dynamic language. Front. Plant Sci. 3 (76), 1–20. 10.3389/fpls.2012.00076 22670147 PMC3362793

[B14] Buckley T. N. Turnbull T. L. Adams M. A. (2012). Simple models for stomatal conductance derived from a process model: cross-validation against sap flux data. Plant Cell Environ. 35, 1647–1662. 10.1111/j.1365-3040.2012.02515.x 22486530

[B15] Campbell G. S. Norman J. M., (1998). An introduction to environmental biophysics. 2nd edition New York: Springer-Verlag, 286. 10.1007/978-1-4612-1626-1

[B16] Cescatti A. (1997). Modelling radiative transfer in discontinuous canopies of asymmetric crowns. I. Model structure and algorithms. Ecol. Model. 101, 263–274. 10.1016/S0304-3800(97)00050-1

[B17] Churkina G. Schimel D. Braswell B. H. Xiao X. (2005). Spatial analysis of growing season length control over net ecosystem exchange. Global Change Biol. 11 (10), 1777–1787. 10.1111/j.1365-2486.2005.001012.x

[B18] Dauzat J. Franck N. Rapidel B. Luquet D. Vaast P. (2007). “Simulation of echophysiological processes on 3D virtual stands with the ARCHIMED simulation platform,” in Second international symposium on plant growth modeling, simulation, visualization and applications. (Beijing, China: IEEE), 101–108. 10.1109/PMA.2006.52

[B19] De Pury D. G. G. Farquhar G. D. (1997). Simple scaling of photosynthesis from leaves to canopies without the errors of big-leaf models. Plant Cell Environ. 20, 537–557. 10.1111/j.1365-3040.1997.00094.x

[B20] Duursma R. A. Medlyn B. E. (2012). MAESPA: a model to study interactions between water limitation, environmental drivers and vegetation function at tree and stand levels, with an example application to [CO_2_]× drought interactions. Geosci. Model Dev. 5, 919–940. 10.5194/gmd-5-919-2012

[B21] Evers J. B. Letort V. Renton M. Kang M. (2018). Computational botany: advancing plant science through functional-structural plant modelling. Ann. Bot. 121, 767–772. 10.1093/aob/mcy050

[B22] Farquhar G. D. von Caemmerer S. Berry J. A. (1980). A biochemical model of photosynthetic CO_2_ assimilation in leaves of C_3_ species. Planta 149, 78–90. 10.1007/BF00386231 24306196

[B23] Friend A. (2001). Modelling canopy CO_2_ fluxes: are ‘big-leaf’ simplifications justified? Global Ecol. Biogeo. 10, 603–619. 10.1046/j.1466-822x.2001.00268.x

[B24] Gastellu-Etchegorry J.-P. Grau E. Lauret N. (2012). DART: a 3D model for remote sensing images and radiative budget of earth images. In Modeling and simulation in engineering. Ed. Alexandru C. (Rijeka, Croatia: InTech), 29–68.

[B25] Ginzburg L. R. Jensen C. X. J. (2004). Rules of thumb for judging ecological theories. Trends Ecol. Evol. 19, 121–126. 10.1016/j.tree.2003.11.004 16701242

[B26] Gueymard C. A. (2003). Direct solar transmittance and irradiance predictions with broadband models. Part I: detailed theoretical performance assessment. Solar Energy 74, 355–379. 10.1016/S0038-092X(03)00195-6

[B27] Hemmerling R. Kniemeyer O. Lanwert D. Kurth W. Buck-Sorlin G. (2008). The rule-based language XL and the modelling environment GroIMP illustrated with simulated tree competition. Funct. Plant Biol. 35, 739–750. 10.1071/FP08052 32688828

[B28] Henke M. Buck-Sorlin G. H. (2018). Using a full spectral raytracer for calculating light microclimate in functional-structural plant modelling. Comput. Inf. 36, 1492–1522. 10.4149/cai_2017_6_1492

[B29] Henke M. Kurth W. Buck-Sorlin G. H. (2016). FSPM-P: towards a general functional-structural plant model for robust and comprehensive model development. Front. Comp. Sci. 10, 1103–1117. 10.1007/s11704-015-4472-8

[B30] Holzinger A. (2005). Usability engineering methods for software developers. Commun. ACM 48, 71–74. 10.1145/1039539.1039541

[B31] Iqbal M. (2012). An introduction to solar radiation. Burlington: Elsevier Science.

[B32] Jarvis P. G. McNaughton K. (1986). “Stomatal control of transpiration: scaling up from leaf to region,” in Advances in ecological research, vol. 15 (London, U.K.: Academic Press), 1–49. 10.1016/S0065-2504(08)60119-1

[B33] Johnson I. R. (2010). PlantMod: exploring the physiology of plant canopies. Dorrigo, NSW, Australia: Tech. rep., IMJ Software. URL www.imj.com.au/software/plantmod.

[B34] Kahlen K. Stützel H. (2011). Modelling photo-modulated internode elongation in growing glasshouse cucumber canopies. New Phytol. 190, 697–708. 10.1111/j.1469-8137.2010.03617.x 21251000

[B35] Karwowski R. Prusinkiewicz P. (2003). Design and implementation of the L+C modeling language. Electron. Notes Theor. Comput. Sci. 86, 134–152. 10.1016/S1571-0661(04)80680-7

[B36] Lawrence D. Fisher R. Koven C. Oleson K. Swenson S. Vertenstein M. (2019). CLM5 documentation. Tech. rep., Boulder, CO: National Center for Atmospheric Research.

[B37] Lewis P. (1999). Three-dimensional plant modelling for remote sensing simulation studies using the Botanical Plant Modelling System. Agronomie 19, 185–210. 10.1051/agro:19990302

[B38] Marschner S. Shirley P. (2015). Fundamentals of computer graphics. (Boca Raton, FL: A K Peters/CRC Press), 748.

[B39] Meyers T. P. Paw U K. T. (1987). Modelling the plant canopy micrometeorology with higher-order closure principles. Agric. For. Meteorol. 41, 143–163. 10.1016/0168-1923(87)90075-X

[B40] Mott K. A. Buckley T. N. (2000). Patchy stomatal conductance: emergent collective behaviour of stomata. Trends Plant Sci. 5 (6), 258–262. 10.1016/S1360-1385(00)01648-4 10838617

[B41] Pearcy R. W. Yang W. (1996). A three-dimensional crown architecture model for assessment of light capture and carbon gain by understory plants. Oecologia 108, 1–12. 10.1007/BF00333208 28307727

[B42] Ponce de León M. A. Bailey B. N. (2019). Evaluating the use of Beer’s law for estimating light interception in canopy architectures with varying heterogeneity and anisotropy. Ecol. Model. 406, 133–143. 10.1016/j.ecolmodel.2019.04.010

[B43] Pradal C. Dufour-Kowalski S. Boudon F. Fournier C. Godin C. (2008). OpenAlea: a visual programming and component-based software platform for plant modelling. Funct. Plant Biol. 35, 751–760. 10.1071/FP08084 32688829

[B44] Prata A. J. (1996). A new long-wave formula for estimating downward clear-sky radiation at the surface. Q.J.R. Meteorol. Soc. 122, 1127–1151. 10.1002/qj.49712253306

[B45] Press W. H. Teukolsky S. A. Vetterling W. T. Flannery B. P. (2007). Numerical recipes: the art of scientific computing. (Cambridge, U.K.: Cambridge University Press), 1256.

[B46] Prusinkiewicz P. Runions A. (2012). Computational models of plant development and form. New Phytol. 193, 549–569. 10.1111/j.1469-8137.2011.04009.x 22235985

[B47] Raupach M. Finnigan J. (1988). ‘Single-layer models of evaporation from plant canopies are incorrect but useful, whereas multilayer models are correct but useless’: discuss. Aust. J. Plant Physiol. 15, 705–716. 10.1071/PP9880705

[B48] Reichstein M. Falge E. Baldocchi D. Papale D. Aubinet M. Berbigier P. (2005). On the separation of net ecosystem exchange into assimilation and ecosystem respiration: review and improved algorithm. Global Change Biol. 11 (9), 1424–1439. 10.1111/j.1365-2486.2005.001002.x

[B49] Ross J. (1981). The radiation regime and architecture of plant stands. The Hague, The Netherlands: Dr. W. Junk Publishers, 424. 10.1007/978-94-009-8647-3

[B50] Sellers P. Mintz Y. Sud Y. Dalcher A. (1986). A simple biosphere model (SiB) for use within general circulation models. J. Atmos. Sci. 43, 505–531. 10.1175/1520-0469(1986)043<0505:ASBMFU>2.0.CO;2

[B51] Sinclair T. R. Murphy C. E. Knoerr K. R. (1976). Development and evaluation of simplified models for simulating canopy photosynthesis and transpiration. Brit. Ecol. Soc. 13, 813–829. 10.2307/2402257

[B52] Sinoquet H. Le Roux X. Adam B. Ameglio T. Daudet F. A. (2001). RATP: a model for simulating the spatial distribution of radiation absorption, transpiration and photosynthesis within canopies: application to an isolated tree crown. Plant Cell Environ. 24, 395–406. 10.1046/j.1365-3040.2001.00694.x

[B53] Suffern K. G. (2007). Ray tracing from the ground up. (Boca Raton, FL: A K Peters/CRC Press), 784.

[B54] Valladares F. (2003). “Light heterogeneity and plants: from ecophysiology to species coexistence and biodiversity,” in Progress in botany (Berlin Heidelberg: Springer-Verlag), 439–471. 10.1007/978-3-642-55819-1_17

[B55] Vezy R. Christina M. Rouspard O. Nouvellon Y. Duursma R. Medlyn B. (2018). Measuring and modelling energy partitioning in canopies of varying complexity using MAESPA model. Agric. For. Meteorol. 253-254, 203–217. 10.1016/j.agrformet.2018.02.005

[B56] Viswanadham Y. (1981). The relationship between total precipitable water and surface dew point. J. Appl. Meteorol. 20, 3–8. 10.1175/1520-0450(1981)020<0003:TRBTPW>2.0.CO;2

[B57] Wang Y. P. Jarvis P. G. (1990). Description and validation of an array model—MAESTRO. Agric. For. Meteorol. 51, 257–280. 10.1016/0168-1923(90)90112-J

[B58] Wang Y. P. Leuning R. (1998). A two-leaf model for canopy conductance, photosynthesis and partitioning of available energy I: model description and comparison with a multi-layered model. Agric. For. Meteorol. 91, 89–111. 10.1016/S0168-1923(98)00061-6

[B59] Weber J. Penn J. (1995). “Creation and rendering of realistic trees,” in SIGGRAPH ‘95 Proceedings of the 22nd annual conference on computer graphics and interactive techniques (New York: ACM), 119–128. 10.1145/218380.218427

[B60] Woods H. A. Saudreau M. Princebourde S. (2018). Structure is more important than physiology for estimating intracanopy distributions of leaf temperatures. Ecol. Evol. 8, 5206–5218. 10.1002/ece3.4046 29876095 PMC5980536

